# Combined analysis of bulk and single-cell RNA sequencing reveals novel natural killer cell-related prognostic biomarkers for predicting immunotherapeutic response in hepatocellular carcinoma

**DOI:** 10.3389/fimmu.2023.1142126

**Published:** 2023-03-28

**Authors:** Kai Zhang, Enwu Yuan

**Affiliations:** Department of Laboratory Medicine, Third Affiliated Hospital of Zhengzhou University, Zhengzhou, Henan, China

**Keywords:** NK, biomarker, HCC, immunotherapy, TUBA1B

## Abstract

**Introduction:**

Natural killer (NK) cells play an irreplaceable and important role as a subtype of innate immune cells in the contemporary setting of antitumor immunity.

**Methods:**

We chose a total of 1,196 samples for this analysis from the public dataset’s six separate cohorts. To identify 42 NK cell marker genes, we first carried out a thorough study of single-cell RNA sequencing data from the GSE149614 cohort of hepatocellular carcinoma (HCC).

**Results:**

Using the NK cell marker genes in the TCGA cohort, we next created a seven-gene prognostic signature, separating the patients into two categories with distinct survival patterns. This signature’s prognostic prediction ability was well verified across several validation cohorts. Patients with high scores had higher TIDE scores but lower immune cell infiltration percentages. Importantly, low-scoring patients had superior immunotherapy response and prognosis than high-scoring patients in an independent immunotherapy cohort (IMvigor210). Finally, we used CD56 and TUBA1B antibodies for immunohistochemical labeling of HCC tissue sections, and we discovered a lower number of CD56+ cells in the HCC tissue sections with high TUBA1B expression.

**Discussion:**

In summary, our research created a unique prognostic profile based on NK cell marker genes that may accurately predict how well immunotherapy would work for HCC patients.

## 1 Introduction

It is generally recognized that a diverse tumor microenvironment (TME) surrounds tumor cells in hepatocellular carcinoma (HCC) (1). The TME, which has a very complicated makeup, is crucial to the development and growth of tumors. Additionally, the interaction between immune cells and tumor cells in the TME not only influences a patient’s prognosis but may also alter a patient’s response to immunotherapy (2). The importance of innate immune cells has not gotten enough attention in the contemporary setting of antitumor immunity, which has mostly focused on adaptive T-cell responses, like CD4+ CD25+ Foxp3 regulatory T cells (Tregs), cytotoxic T lymphocytes (CTLs) et all (3, 4). By specifically identifying and eliminating tumor cells and encouraging adaptive T-cell immunity responses, natural killer (NK) cells are a subtype of innate immune cells that can reduce the proliferative and invasive potential of tumor cells at an early tumor stage (5). The balance of inhibitory and activating receptors that can interact with ligands on target cells determines how well NK cell function. NK cells can collaborate with T cells to control the spread of cancer and play a crucial part in the development of antitumor immunity. Cancer risk is increased by decreased NK cell activity in peripheral blood (6). Additionally, more tumor-infiltrating NK cells are strongly linked to improved prognosis across a variety of tumor types (7). Given the function of NK cells in immunity, earlier research has focused on their molecular features in cancer and infectious disorders (8, 9), but little is known about their complete molecular analysis in HCC.

Unprecedented chances to unveil the molecular properties of various immune cell populations in TME have been made possible by the advent of single-cell RNA sequencing (scRNA-seq) technology and related data processing methodologies (10). The prognosis and immunotherapeutic response in cancer patients may be accurately predicted by examining gene expression patterns based on molecular characterization of immune cells acquired from scRNA-seq data, according to previous research (11, 12). In this work, we first carried out a thorough examination of scRNA-seq data in HCC to characterize the molecular properties of tumor-infiltrating NK cells and to identify NK cell flag genes. Then, using bulk RNA-seq analysis, NK cell marker gene-related signatures for predicting the prognosis of HCC were created. Additionally, the link between NKCMGS and HCC immunotherapy response was examined, and the predictive ability of NKCMGS was verified in three separate cohorts from the ICGC and the Gene Expression Omnibus (GEO) database. Multiple datasets from TCGA, GEO and ICGC cohort were analyzed in our study for constructed NK cell-related genetic signature. We obtained a more parsimonious gene signature over existing studies, which contains seven genes, and provided better prediction for immunotherapeutic effect and drug sensitivity.

## 2 Methods

### 2.1 Data collection and pre-processing

A total of 1196 samples, 31396 cells, including 10 HCC samples with single-cell RNA-sequencing (scRNA-seq) data from the GSE149614 cohort, 342 HCC samples from the Cancer Genome Atlas (TCGA) cohort (https://xenabrowser.net/), 230 HCC samples from the International Cancer Genome Consortium (ICGC) cohort (https://dcc.icgc.org/), 221 HCC samples from the GSE14520 cohort, 95 HCC samples from the GSE76427 cohort, and 298 samples treated with immunotherapy from the IMvigor210 cohort (http://research-pub.Gene.com/imvigor210corebiologies/), were enrolled in this study. In the GSE149614 dataset, with each gene expressed in at least three cells and each cell expressing more than 250 genes, single cells were initially screened for scRNA-seq data. The percentage of mitochondria and rRNA was then calculated by the Seurat package (13). Further testing of the single cells involved caused each one to express at least 5000 genes with a UMI > 100. The mitochondrial content was no more than 30%. In the end, 31396 cells were still present for identifying the NK cell marker genes of HCC. To find survival-related genes and create prognostic signatures, the bulk transcriptome data (FPKM normalized) and clinical details of HCC patients in the TCGA were employed. For external validation, three separate datasets were acquired: ICGC, GSE14520, and GSE76427.

### 2.2 Identification of NK cells and their hub genes

The GSE149614 dataset contains scRNA-seq data from 10 HCC samples, which we again examined. Following log normalization of the expressed genes, uniform flow-form approximation and projection techniques were used to reduce nonlinear dimensionality. We used the FindNeighbors and FindClusters () algorithms to arrange individual cells into 17 separate subgroups at dim=50 and resolution=0.1. Three marker genes, including CD3D, CD3E, and NKG7, were identified in NK cells. Using the FindAllMarkers program with logFC=0.5, minpct=0.25, and adjusted p-values less than 0.05, marker genes were found for each NK subpopulation. A univariate Cox regression analysis with P less than 0.05 was then used to further identify the genes among these NK marker genes that are associated with prognosis. We used the LASO-Cox regression to compress the number of genes and created a risk profile based on the outcomes of the multivariate Cox model using the equation: Risk score =∑iCoefficient (Genei)*Expression (Genei). Depending on their risk assessments, patients were separated into high- and low-risk groups. The receiver operating characteristic curve (ROC) analysis and Kaplan-Meier survival analysis were used to examine the risk profile’s ability to predict survival outcomes. The validation cohort underwent a similar examination. GSEA was used to examine KEGG, GO, and HALLMARK elements that had drastically changed across the different categories.

### 2.3 Analysis of the immune landscape

Based on the gene expression patterns of HCC patients, the stromal and immune scores were computed using the ESTIMATE software (14). The abundance ratio of immune cells was evaluated using the CIBERSORT (15), MCPcounter (16), and TIMER (17) databases to learn more about the TME.

### 2.4 Response to immune checkpoint blockade (ICB) and analysis of the sensitivity to potential therapeutic drugs

The TIDE database (http://tide.dfci.harvard.edu/), which estimates how often immunotherapy for HCC patients will be effective, was first performed. We also retrieved the matched clinical and transcriptome data from the IMvigor210 cohort of patients who were receiving anti-PD-L1 medication. Additionally, we evaluated multiple immune checkpoint gene expression changes such as PD1, PD-L1, CTLA4, and PD-L2 in different subgroups. Finally, we searched the Cellminer database for delicate medications that successfully address this risk profile (18). If a drug’s adjusted P-value was less than 0.001 and its Pearson correlation coefficient was larger than 0.3, it was categorized as tumor-sensitive. The discrepancies in the half-maximal inhibitory doses (IC50) of several classes of tumor-sensitive medications were then studied.

### 2.5 Evaluation of TUBA1B expression in clinical samples

Using 30 samples of HCC and related paracancerous tissues that had undergone standard pathological evaluation in our pathology department, a validation cohort was developed. The clinicopathological information for all patients were shown in **Table 1**. Following the tissue wax blocks’ serial sectioning, sample sections were gathered and stored for subsequent use in a 4°C freezer. We next used TUBA1B (Abcam, ab108629) and CD56 (Abcam, ab75813) antibodies in IHC experiments on formalin-fixed de-paraffinized slices and captured pictures using microscopy as previously reported (19).

**Table 1:** Characteristics of patients and tumor samples studied (n=30).

Clinicopathological characteristic	
Age, median (range) Female	56.5 (38–74) years old12/30 (40%)
T stage of primary tumor	
T1	2/30 (6.67%)
T2	5/30 (16.67%)
T3	16/30 (53.33%)
T4	7/30 (23.33%)
N stage of primary tumor	
N0	6/30 (20%)
N1	15/30 (50%)
N2	9/30 (30%)
Lymphovascular invasion present in primary tumor	17/30 (56.67%)
Perineural invasion present in primary tumor	11/30 (36.67%)
Synchronous metastasis (unknown for n=2)	13/28 (46.43%)
Underlying liver disease etiology (unknown for n=6)	
HBV, HCV and hepatocirrhosis	14/24 (58.33%)
Fatty liver and diabetes mellitus	4/24 (16.67%)
A**lcohol** Hereditary liver cancer	5/24 (20.83%) 1/24 (4.17%)

### 2.6 Statistical analysis

R software was used to conduct all statistical analyses (v4.1.2). Pearson correlation was used to calculate the correlation analysis. For comparisons between the two groups, the chi-square test and grouped t-test were used, respectively. Kaplan-Meier survival analysis and a Log-rank test were used to assess survival differences between groups. Using the RMS software, a nomogram was produced following the signature. Statistics were deemed significant when the P value was less than 0.05.

## 3 Results

### 3.1 Identification of NK cells in the scRNA-seq samples

**Figure 1:**
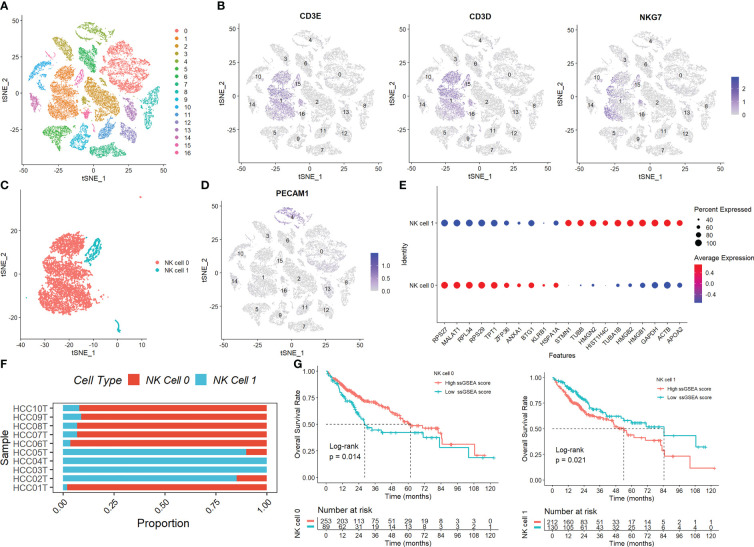
Identification of NK cells in the scRNA-seq samples. **(A)** The TSNE plots showed the distribution of the 17 clusters. **(B)** The expression of three marker genes (CD3D, CD3E, and NKG7) in the 17 clusters. **(C)** Two NK cell subsets were discovered. **(D)** The expression of PECAM1 gene. **(E)** The expression of the top 10 DEGs in the two clusters. **(F)** The percentage of the two clusters in each sample. **(G)** The samples in the high ssGSEA score group in the NK 0 cluster had a better prognosis than those in the low ssGSEA score group, while the opposite finding was seen in the NK 1 cluster.

**Figure 2:**
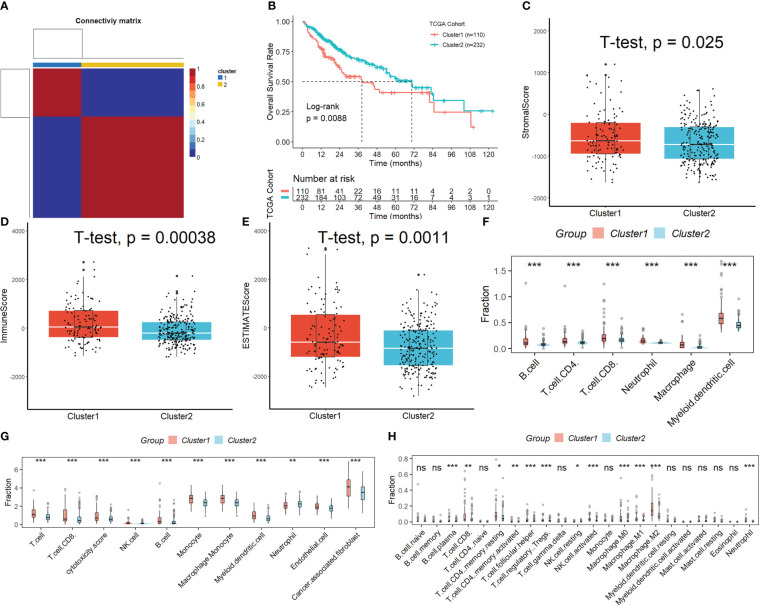
Subtypes identification by NMF algorithm based on NKs marker genes. **(A)** Samples were split into two subclasses. **(B)** Significant disparities in patient survival existed between the two subgroups. **(C-E)** Difference of immunological, stromal, and ESTIMATE scores in different molecular subtypes. The difference in the percentage of immune cell infiltration in different molecular subtypes was analyzed by the TIMER **(F)**, MCPcounter **(G)**, and CIBISORT **(H)**. ns, not significant; *p < 0.05; **p <0.01; ***p < 0.001.

**Figure S1** displays the full outcomes of data preparation. After log-normalization and dimensionality reduction, 17 clusters were found. The TSNE plots showing the distribution of the 17 clusters are displayed in **Figure 1A**. As shown in **Figure 1B** and S2A, based on the expression of three marker genes (CD3D, CD3E, NKG7, CXCR3 and IL2RB), two NK cell subsets were discovered (**Figure 1C**). The fact that neither of the two NK cell clusters expressed the epithelial cell-specific gene (PECAM1) proves that NKs were correctly identified (**Figure 1D**). Further analysis of CD19 and CD14 expression in 17 clusters was performed for ruled out the interference of other cell types (**Figure S2B**). The expression of the top 10 DEGs in the two clusters is shown in **Figure 1E**. The 2 NK cell clusters contained 42 DEGs (marker genes recognized as NK clusters). The percentage of the two clusters in each sample was shown in **Figure 1F**. Furthermore, we computed the ssGSEA scores for the marker genes of each NK cluster (the top 10 DEGs of the NK clusters) in the TCGA cohort to examine the connection between NK clusters and prognosis. The samples in the high ssGSEA score group in the NK 0 cluster had a better prognosis than those in the low ssGSEA score group, while the opposite finding was seen in the NK 1 cluster (**Figure 1G**). Finally, to further analyze the function and mechanism of NKs marker genes in HCC, we performed molecular subtype identification analysis of the TCGA dataset by non-negative matrix decomposition (NMF) algorithm. The HCC samples were split into two subclasses based on the NKs marker genes after it was found that two clusters were the ideal number (**Figures 2A**, **S3**). Significant disparities in patient survival existed between the two subgroups (**Figure 2B**). Additionally, the two subgroups’ TME features were contrasted. **Figures 2C–E** demonstrates that as compared to samples from cluster 2, samples from cluster 1 had higher immunological, stromal, and ESTIMATE scores. HCC patients in Cluster 1 had a larger percentage of immune cell infiltration in their TME, as shown by the results of the TIMER (**Figure 2F**), MCPcounter (**Figure 2G**), and CIBIS. ORT (**Figure 2H**).

### 3.2 Screening for NK-associated hub genes

**Figure 3:**
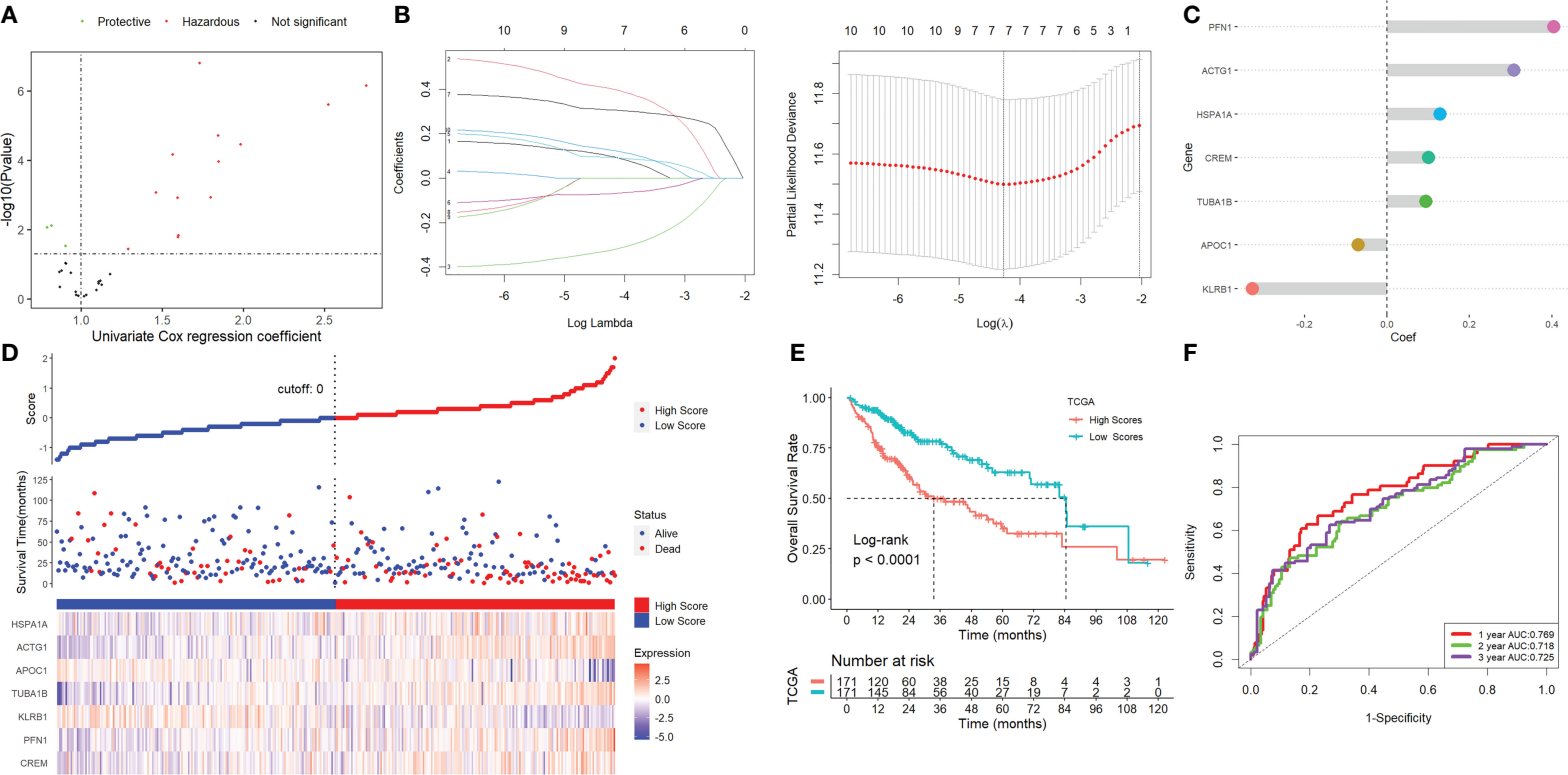
Screening for NK-associated hub genes. **(A)** Using univariate Cox regression analysis. **(B)** Lasso-Cox regression analysis. **(C)** Seven genes were left with a lambda value of 0.0139. **(D)** After each sample’s score was determined, the groups of high- and low-risk individuals were created. **(E)** Kaplan-Meier survival analysis. **(F)** ROC analysis.

**Figure 4:**
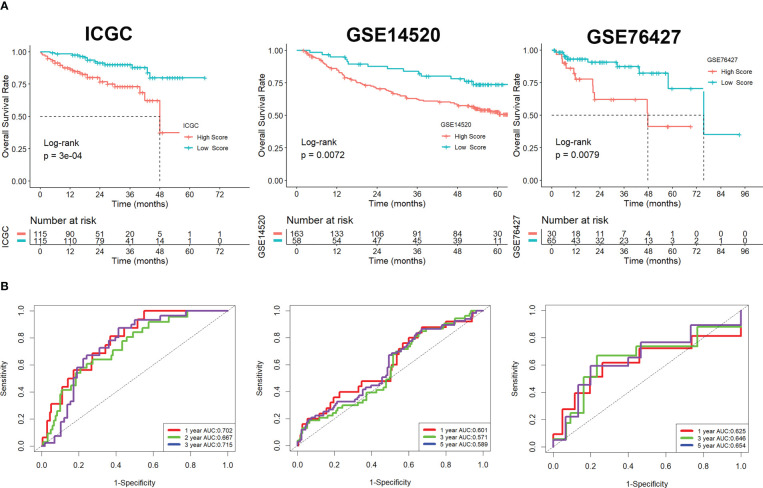
Validation of the signature in the ICGC and GEO cohorts. **(A)** Kaplan-Meier survival analysis. **(B)** ROC analysis.

Using univariate Cox regression analysis, the prognostic value of these DEGs was evaluated to create a signature, with 10 genes displaying prognostic values (**Figure 3A**). To reduce the number of genes, Lasso-Cox regression analysis was used (**Figure 3B**). Seven genes were left with a lambda value of 0.0139 (**Figure 3C**). After multivariate Cox regression analysis, we finally included these seven genes (CREM, PFN1, KLRB1, TUBA1B, APOC1, ACTG1, and HSPA1A) in the signature. Following is the final seven-gene signature formula: Score = (0.1574×CREM) + (0.4582×PFN1) - (0.3804×KLRB1)+(0.1094×TUBA1B)-(0.0821×APOC1)+(0.3267×ACTG1) + (0.1718×HSPA1A). After each sample’s score was determined, the groups of high- and low-risk individuals were created (**Figure 3D**). The association between score and clinical characteristics was first evaluated and found that higher scores were associated with HBV infection, advanced TNM stage, later grade, later T stage, and recurrence (**Figure S4**). In both the TCGA, ICGC, and the GEO cohort, Kaplan-Meier survival analyses showed that high-risk patients had considerably worse survival outcomes than low-risk patients (**Figures 3E**, **4A**). The TCGA cohort’s AUC values for the model for 1- to 3-year survival range from 0.71 to 0.77 (**Figures 3F**), whereas those for the ICGC and GEO cohorts ranged from 0.57 to 0.72 (**Figures 4B**).

### 3.3 Mutation and functional enrichment analysis of the signature

The results of Gene Set Enrichment Analysis (GSEA) revealed that the majority of the impacted HALLMARK, GO, and KEGG components were engaged in DNA synthesis and replication, mitosis, chromosome segregation, and other biological processes related to the cell cycle (**Figure S5**). Studies on genetic modification that focused on significantly altered genes showed that the mutation rates in the two groups were very different from one another (**Figure S6A**). After tumor mutational burden (TMB) values for each HCC patient were analyzed, we found that patients in the high-score group with greater TMB values had the lowest overall survival rates, while the opposite results were found in patients in the low-score group with lower TMB values (**Figure S6B**).

### 3.4 Correlation analysis between the signature and immunity

**Figure 5:**
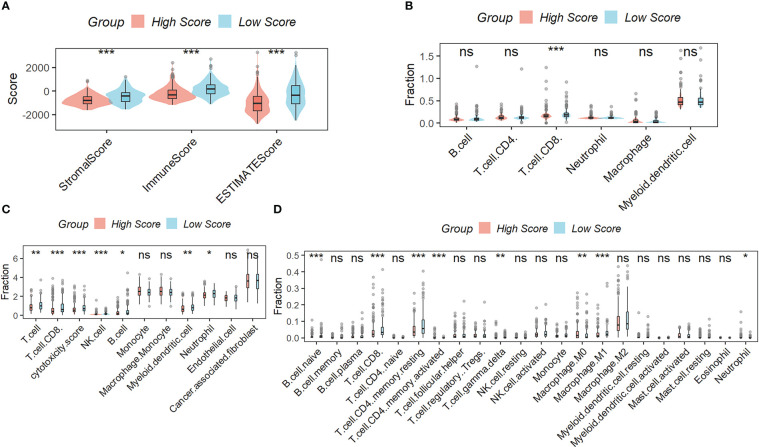
Correlation Analysis between the signature and immunity. **(A)** samples in the low-scoring group had higher immune, stromal, and ESTIMATE scores compared to samples in the high-scoring group. The difference in the percentage of immune cell infiltration in different molecular subtypes was analyzed by the TIMER **(B)**, MCPcounter **(C)**, and CIBISORT **(D)**. ns, not significant; *p < 0.05; **p <0.01; ***p < 0.001.

**Figure 5A** showed that samples in the low-scoring group had higher immune, stromal, and ESTIMATE scores compared to samples in the high-scoring group. As shown in the results of TIMER (**Figure 5B**), MCPcounter (**Figure 5C**), and CIBISORT (**Figure 5D**), a greater percentage of immune cell infiltration was found in the TME of HCC patients in the low-scoring group.

### 3.5 The signature’s response to PD-L1 blockade immunotherapy

**Figure 6:**
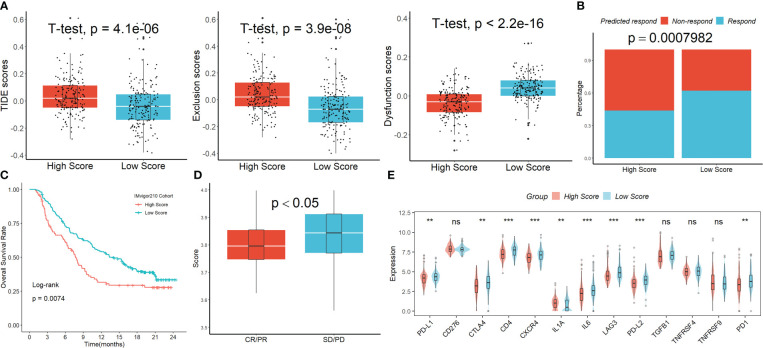
The signature’s response to PD-L1 blockade immunotherapy. **(A)** The TIDE analysis. **(B)** When the projected immunotherapy response rate was included, the proportion of “respond” was lower in the high-risk group. **(C)** In comparison to the high-scoring group, patients in the low-scoring group saw notable clinical benefits and considerably longer overall life in the IMvigor210 cohort. **(D)** Patients with PD/SD had greater risk ratings than those who had a complete response CR/PR. **(E)** The expression levels of the ICIs genes in the two groups. ns, not significant; **p <0.01; ***p < 0.001.

The Tumor Immune Dysfunction and Exclusion (TIDE) analysis showed that, although the exclusion scores had the opposite impact, the TIDE and dysfunction scores were significantly greater in the group with higher risk scoring than in the group with lower risk scoring (**Figure 6A**). When the projected immunotherapy response rate was included, the proportion of “respond” was lower in the high-risk group (**Figure 6B**). Then, using data from the IMvigor210 cohort, we assessed the predictive efficacy of immune checkpoint treatment risk factors. In comparison to the high-scoring group, patients in the low-scoring group saw notable clinical benefits and considerably longer overall life (**Figure 6C**). **Figure 6D** showed that patients with progressing disease (PD) or stable disease (SD) had greater risk ratings than those who had a complete response (CR)/partial response (PR). Finally, we discovered that the levels of the genes PD-1, PD-L1, PD-L2, CTLA4, CD4, CXCR4, LAG3, and LL6 were higher in patients with lower scores than in patients with higher scores (**Figure 6E**), suggesting that these ICIs may be more beneficial for patients with lower scores.

### 3.6 Construction of a nomogram model and exploration of potential drug sensitivity

As shown in **Figure S7A**, we created a nomogram incorporating clinical features and the signature to maximize the predictive performance of risk characteristics. The calibration plots demonstrated that the nomogram was capable of accurately forecasting the final survival rate (**Figure S7B**). In addition, we identified 7 drugs with tumor sensitivity (**Figure S8A**). As shown in **Figure S8B**, we also found that the IC50 for Cladribine, Fludarabine, and Clofarabine was lower in patients with higher scores (20–22).

### 3.7 TUBA1B expression in HCC

**Figure 7:**
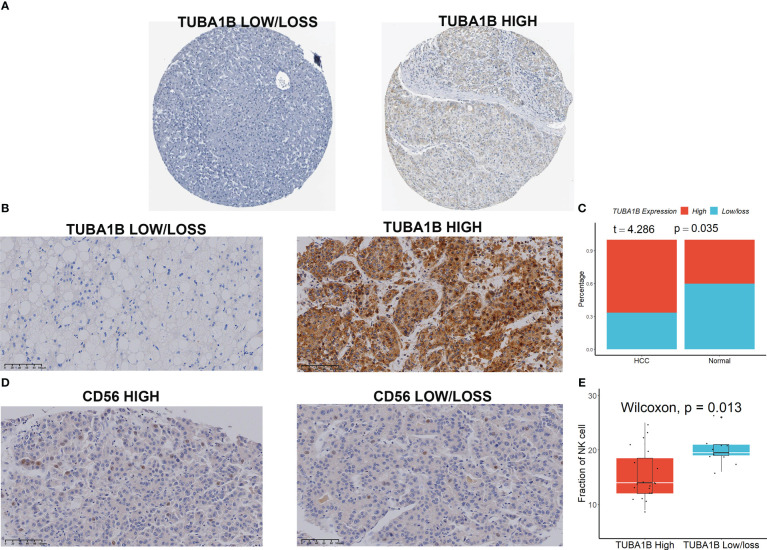
TUBA1B expression in HCC. **(A)** TUBA1B expression explored in HPA database. **(B-C)** TUBA1B expression explored by IHC in clinical samples. **(D)** CD56 expression explored by IHC in HCC samples. **(E)** A lower number of CD56-positive cells in the HCC tissue sections with high TUBA1B expression.

We first initially investigated the expression and prognostic value of these seven genes in HCC in the GEPIA database (23). We found that only TUBA1B showed differential expression in HCC and normal tissues (**Figure S9A**), although several genes including KLRB1, TUBA1B, APOC1, ACTG1, and HSPA1A had high prognostic values (**Figure S9B**). We then focused our main attention on TUBA1B. Using the Human Protein Atlas database (HPA) (24), we found significant variability in the protein expression of TUBA1B in normal and HCC tissues (**Figure 7A**). This phenomenon was confirmed in clinical HCC and normal tissue sections (**Figures 7B, C**). Last but not least, we found differential expression of CD56 in these HCC tissues (**Figure 7D**) and a lower number of CD56-positive cells in the HCC tissue sections with high TUBA1B expression (**Figure 7E**).

## 4 Discussion

Researchers are learning more about the variety and heterogeneity of TME as well as the molecular properties of tumor-infiltrating immune cells thanks to the quick development of scRNA-seq technology (25). However, the majority of recent research has concentrated on adaptive immune cells, and the function of innate immune cells has not received enough attention, which may have a significant impact on the prognosis and effectiveness of immunotherapy in patients with tumors (26). If neighboring cells exhibit surface markers linked to oncogenic transformation, NK cells can quickly destroy many of them by improving antibody and T-cell responses (27). The prognosis of patients with various tumors, including lung adenocarcinoma (28), gastric cancer (29, 30), liver cancer (31), melanoma (32), and colorectal cancer (33), is highly correlated with the number of tumor-infiltrating NK cells. The overall survival of HCC following hepatectomy is significantly impacted by the low frequency of NK cells relative to myeloid and other lymphocytes seen in HCC tumor tissue (34). The number of NK cells inside the TME, which has a favorable correlation with patient survival, also influences how well patients respond to sorafenib therapy (35). In the current work, we aimed to investigate NK cell marker genes in HCC by bulk and scRNA-seq analysis as well as to create a transcriptional signature based on NK cell marker genes to evaluate NK cell infiltration in TME. By boosting NK scores, we discovered a substantial classification of HCC patients’ prognosis that was well verified across three separate cohort datasets. Additionally, we discovered that immunotherapy response rates were much greater for patients with low NK scores than for patients with high NK scores, indicating that immune checkpoint blockade treatment is better suitable for individuals with low NK scores.

Immune cells that invade tumors and contribute considerably to tumor growth might have a negative impact on a patient’s prognosis if they have HCC (36). By using the TIMER, MCPcounter, and CIBERSORT algorithms to estimate and compare the abundance of immune cell infiltration between high and low NK score populations, we discovered higher levels of immune cell infiltration, particularly T and B cells, in low NK score tumors, indicating that low NK score tumors are referred to as “hot tumors” with increased antitumor activity (37). The greater survival rate of patients with low NK scores may be partially explained by the strong immune cell infiltration, which may promote tumor cell attenuation to avoid immune monitoring and impede tumor development.

Taking into account that variations in immune cell infiltration between different NK score subgroups affect the effectiveness of immunotherapy that patients receive, we first examined the clinical response to immunotherapy in HCC patients using the Tumor Immune Dysfunction and Exclusion (TIDE) algorithm (38). We found significantly higher TIDE scores in the higher NK score group than in the lower NK score group, and a lower proportion of “respond” in the high NK score group. Subsequently, we validated the predictive power of our NK score with an immunotherapy cohort (Imvigor210). We found that patients with progressing disease (PD) or stable disease (SD) had greater risk ratings than those who had a complete response (CR)/partial response (PR). In light of the possibility that complex TME can cause HCC cells to develop resistance to immune checkpoint inhibitors (ICIs), which could affect the efficacy of immunotherapy, we also looked at the differences in the expression levels of various immune checkpoint genes between high- and low-NK scores subgroups. It has been demonstrated that patients with lower scores had larger prevalences of the genes PD-1, PD-L1, PD-L2, CTLA4, CD4, CXCR4, LAG3, and LL6 than people with higher scores. In conclusion, NK scores may be a valid biomarker for predicting response to immunotherapy, and patients with low NK scores are more likely to benefit from it.

Finally, utilizing the CellMiner database, we discovered seven medicines that are tumor sensitive. Since individuals with higher NK scores had lower IC50s for Cladribine, Fludarabine, and Clofarabine, it is obvious that these patients are more susceptible to these medications. Cladribine and Clofarabine are nucleoside analogs that are frequently used to treat hematologic cancers and target B and T cells (39, 40). Cladribine has been successfully utilized as a first-line therapy for hairy cell leukemia for some time now (41). Unfortunately, when used to treat multiple sclerosis, cladribine can lead to acute, specific liver harm in individuals (42). Additionally, clofarabine is employed as an anticancer therapy for several solid tumors, including bladder (43), stomach (44), and breast malignancies (45). Since fludarabine dramatically reduces the release of HBV progenitor DNA, it has been used to treat HBV infection and enhance the prognosis of HCC that is related to HBV (46). Combining fludarabine with fusion proteins comprising the poliovirus receptor (PVR) and the programmed death-1 (PD-1) extracellular structural domain improves long-term tumor-specific immunosurveillance and CD8+ T cell-mediated anticancer effects (47). However, additional research is required to confirm if these drugs can eventually increase tumor cell death by targeting NK cells.

Despite the encouraging findings, there are several limitations to this study. First, the candidate genes involved in our study were limited to NK cell marker genes, and the tumor immune microenvironment is highly spatially heterogeneous; second, a sizable multicenter randomized controlled trial will be needed in the future to evaluate this signature. Finally, all mechanistic analyses in our study were descriptive. Future studies must explore the potential mechanisms between NK marker gene expression and HCC prognosis.

## 5 Conclusion

In summary, a prognostic seven-gene signature built on NK cell marker genes was discovered and proven to have a strong performance in predicting immunotherapy response in HCC patients. It may be used as a prognostic biomarker to aid in the selection of suitable individuals who would benefit from immunotherapy and support therapeutic decision-making about customized prediction.

## Data availability statement

The datasets presented in this study can be found in online repositories. The names of the repository/repositories and accession number(s) can be found within the article/**Supplementary Materials**.

## Ethics statement

The studies involving human participants were reviewed and approved by Ethics Committees of Zhengzhou University. The patients/participants provided their written informed consent to participate in this study.

## Author contributions

KZ designed this work and analyzed the data; EY performed experiments and analyses; KZ helped for providing tumor samples; KZ wrote the manuscript. All authors contributed to the article and approved the submitted version.
