# Supplementary material for: Combined analysis of bulk and single-cell RNA sequencing reveals novel natural killer cell-related prognostic biomarkers for predicting immunotherapeutic response in hepatocellular carcinoma
Source: Front Immunol. 2023 Mar 28;14:1142126. doi: 10.3389/fimmu.2023.1142126 (PMC10086229; doi:10.3389/fimmu.2023.1142126)
Supplement: Supplementary file 1 [file DataSheet_1.docx]

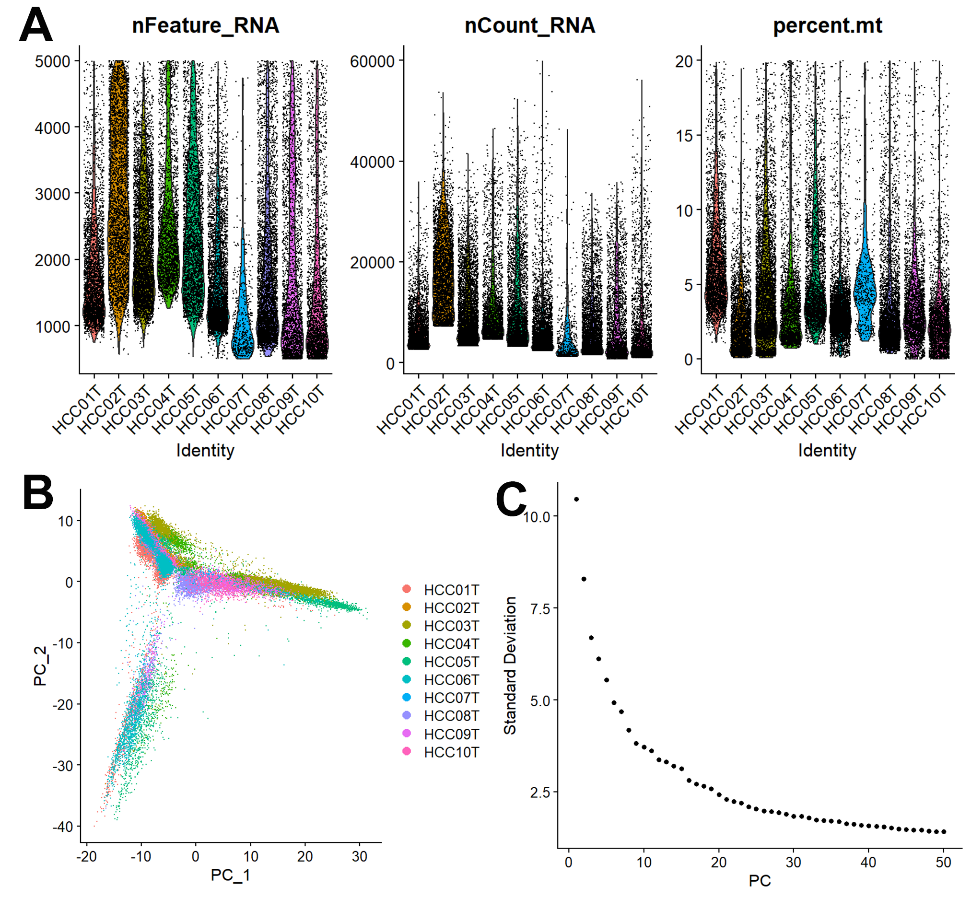


Figure S1 The outcomes of reprocessing the scRNA-seq data in the GSE149614 dataset. (A) After filtering, the correlation between each sample's mRNA, UMI, mitochondrial content, and rRNA content; (B) The sample distribution map of PCA dimensionality reduction; (C) the anchor point map of PCA.


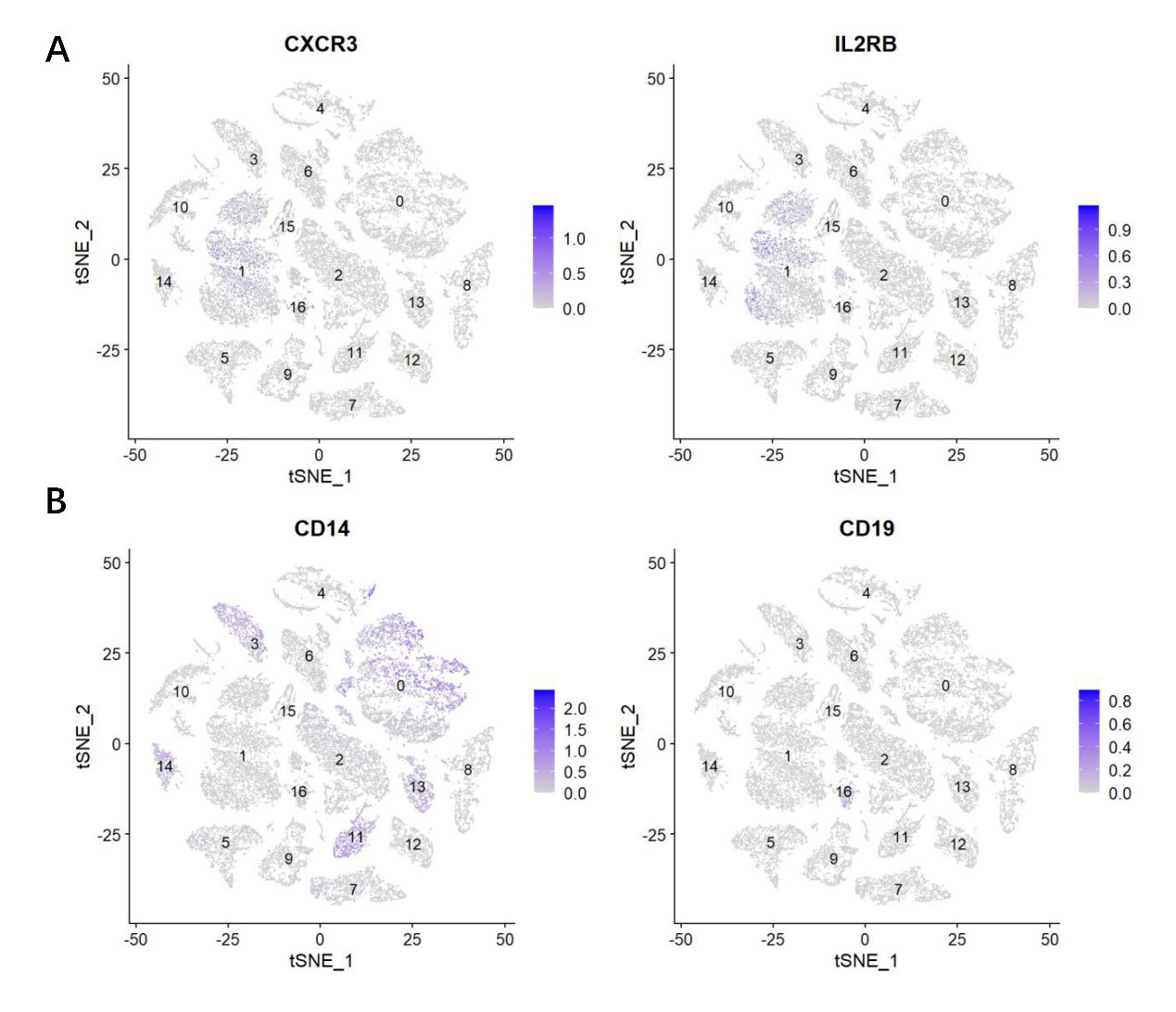


Figure S2 Identification of NK cells in the scRNA-seq samples. (A) The expression of two marker genes for NK cells (CXCR3 and IL2RB) in the 17 clusters. (B) The expression of two marker genes for monocyte and B cells (CD14 and CD19) in the 17 clusters.


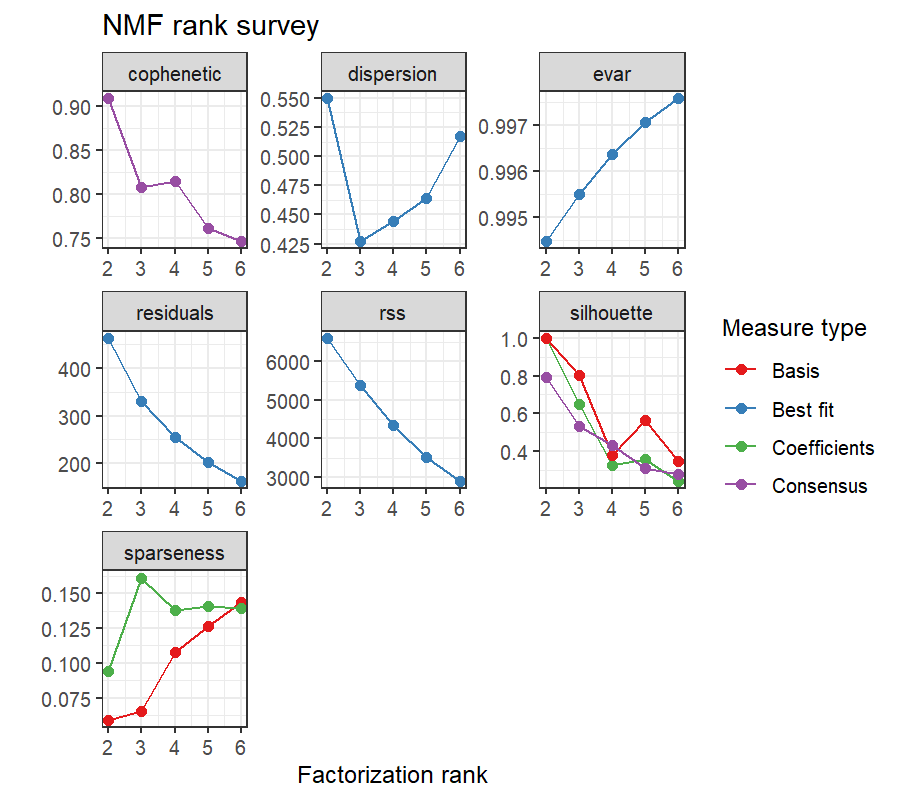


Figure S3 NMF rank survey.


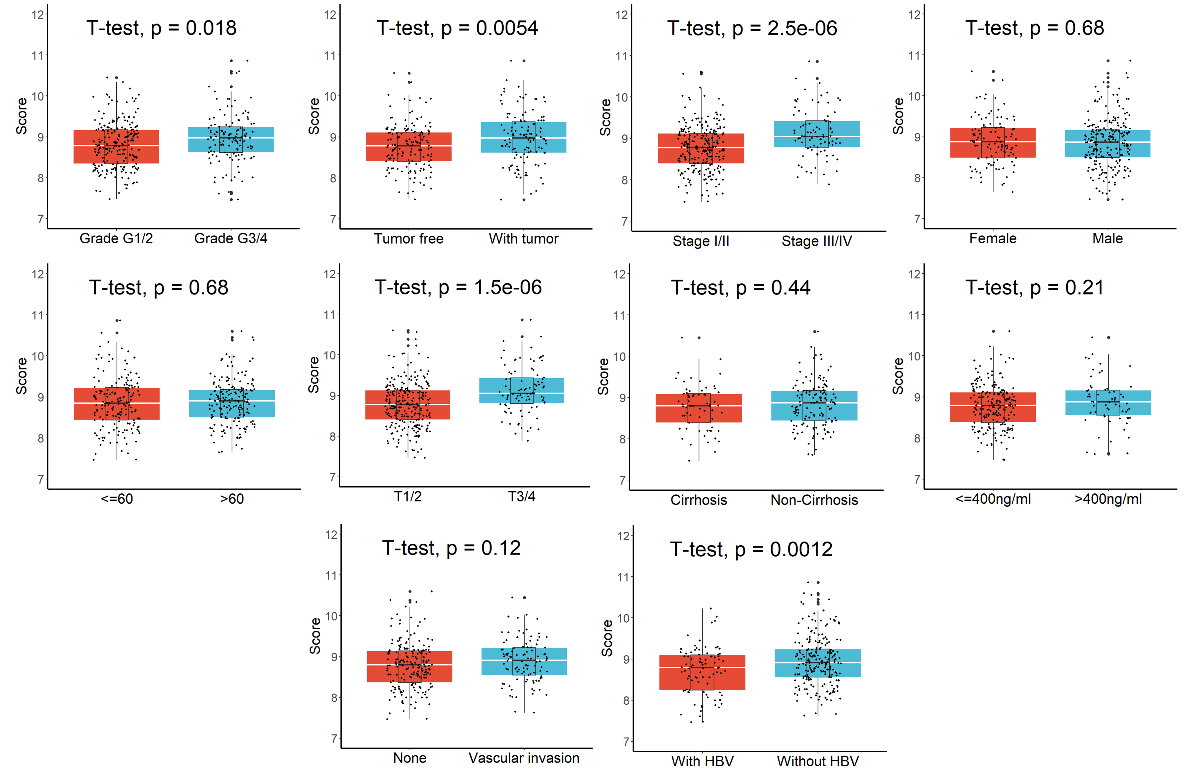


Figure S4 The association between score and clinical characteristics.


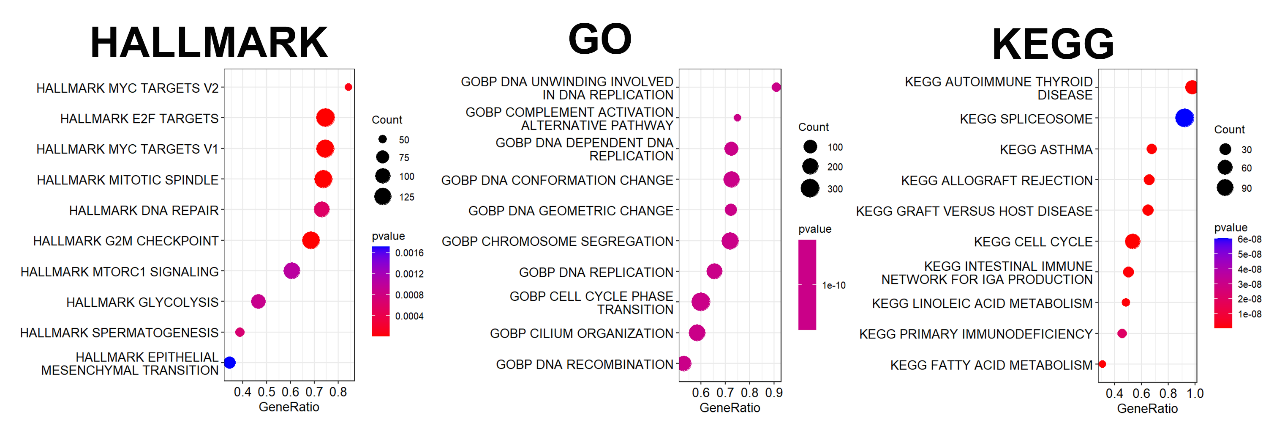


Figure S5 Functional enrichment analysis of the signature.


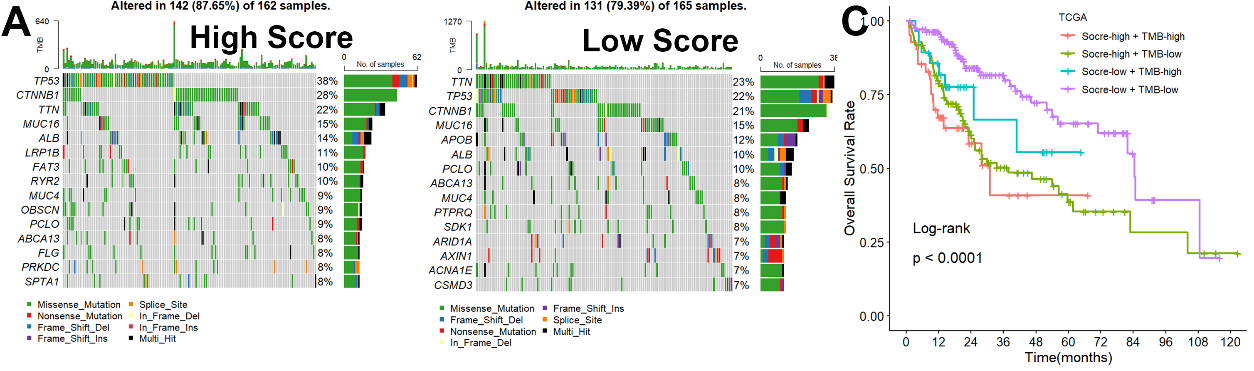


Figure S6 (A) Mutation analysis of the signature. (B) After tumor mutational burden (TMB) values for each HCC patient were analyzed, we found that patients in the high-score group with greater TMB values had the lowest overall survival rates.


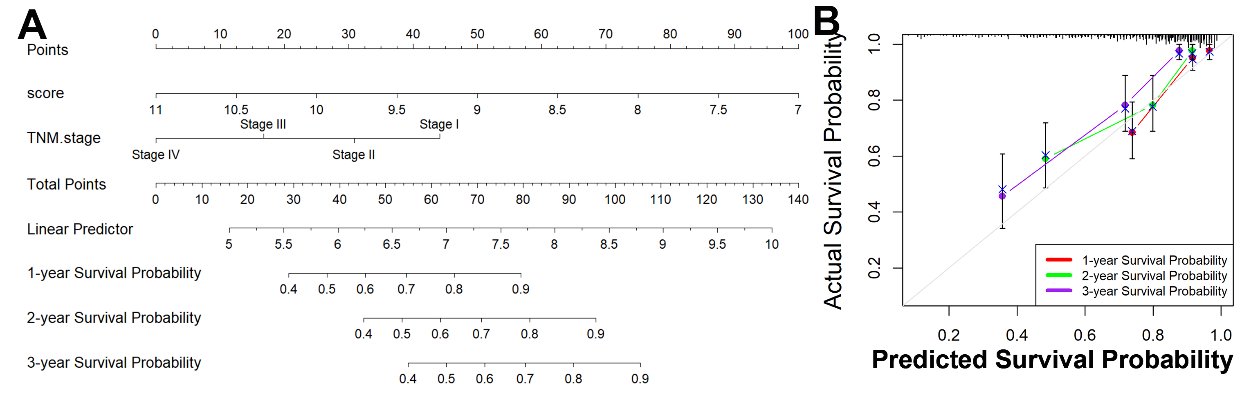


Figure S7 (A) Construction of a nomogram model. (B) The calibration plots.


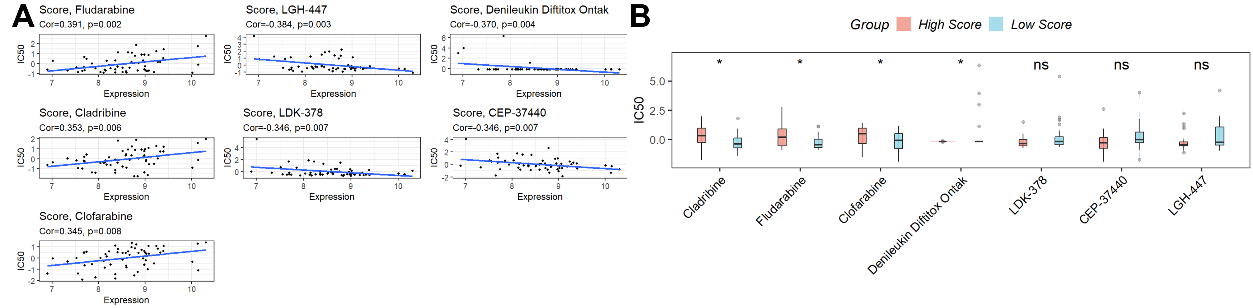


Figure S8 Exploration of potential drug sensitivity. (A) 7 drugs with tumor sensitivity. (B) The IC50 of the 7 drugs. ns, not significant; *p < 0.05.


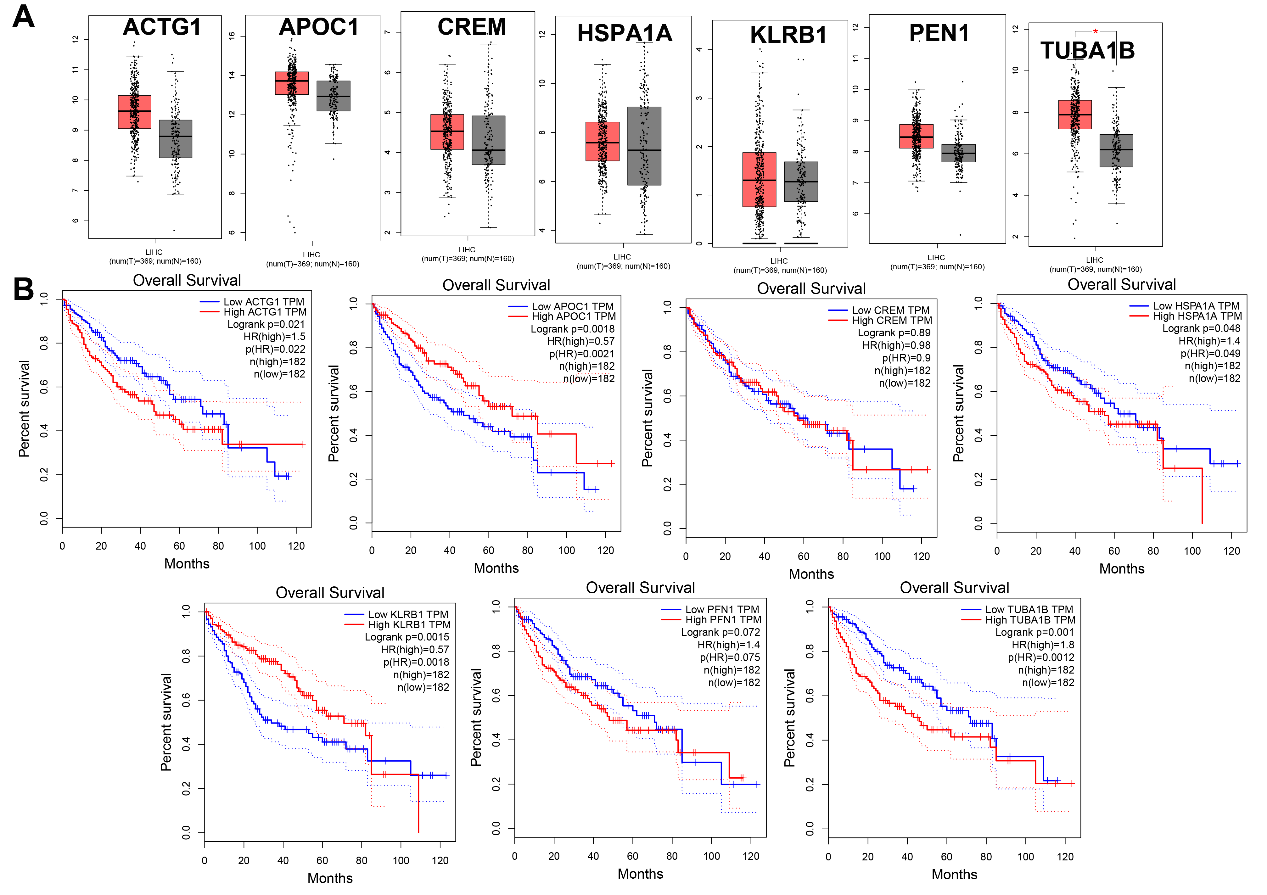


Figure S9 (A) Expression levels and (B) prognostic value of these seven genes in HCC in the GEPIA database.
